# Cryo Electron Tomography of Herpes Simplex Virus during Axonal Transport and Secondary Envelopment in Primary Neurons

**DOI:** 10.1371/journal.ppat.1002406

**Published:** 2011-12-15

**Authors:** Iosune Ibiricu, Juha T. Huiskonen, Katinka Döhner, Frank Bradke, Beate Sodeik, Kay Grünewald

**Affiliations:** 1 Department of Molecular Structural Biology, Max Planck Institute of Biochemistry, Martinsried, Germany; 2 Institute of Biotechnology, University of Helsinki, Helsinki, Finland; 3 Oxford Particle Imaging Centre, Division of Structural Biology, Wellcome Trust Centre for Human Genetics, University of Oxford, Oxford, United Kingdom; 4 Institute of Virology, Hannover Medical School, Hannover, Germany; 5 Research Group Axonal Growth and Regeneration, Max Planck Institute of Neurobiology, Martinsried, Germany; 6 German Center for Neurodegenerative Diseases (DZNE), Bonn, Germany; University of California at Los Angeles, United States of America

## Abstract

During herpes simplex virus 1 (HSV1) egress in neurons, viral particles travel from the neuronal cell body along the axon towards the synapse. Whether HSV1 particles are transported as enveloped virions as proposed by the ‘married’ model or as non-enveloped capsids suggested by the ‘separate’ model is controversial. Specific viral proteins may form a recruitment platform for microtubule motors that catalyze such transport. However, their subviral location has remained elusive. Here we established a system to analyze herpesvirus egress by cryo electron tomography. At 16 h post infection, we observed intra-axonal transport of progeny HSV1 viral particles in dissociated hippocampal neurons by live-cell fluorescence microscopy. Cryo electron tomography of frozen-hydrated neurons revealed that most egressing capsids were transported independently of the viral envelope. Unexpectedly, we found not only DNA-containing capsids (cytosolic C-capsids), but also capsids lacking DNA (cytosolic A-/B-capsids) in mid-axon regions. Subvolume averaging revealed lower amounts of tegument on cytosolic A-/B-capsids than on C-capsids. Nevertheless, all capsid types underwent active axonal transport. Therefore, even few tegument proteins on the capsid vertices seemed to suffice for transport. Secondary envelopment of capsids was observed at axon terminals. On their luminal face, the enveloping vesicles were studded with typical glycoprotein-like spikes. Furthermore, we noted an accretion of tegument density at the concave cytosolic face of the vesicle membrane in close proximity to the capsids. Three-dimensional analysis revealed that these assembly sites lacked cytoskeletal elements, but that filamentous actin surrounded them and formed an assembly compartment. Our data support the ‘separate model’ for HSV1 egress, *i.e.* progeny herpes viruses being transported along axons as subassemblies and not as complete virions within transport vesicles.

## Author Summary

Herpes simplex virus 1 (HSV1) establishes lifelong latent infections in the peripheral nervous system. After reactivation, progeny viral particles travel within sensory neurons towards sites of initial infection. There are conflicting reports what type of viral structures are transported: some studies observed non-enveloped capsids traveling while others reported transport of fully enveloped viruses within vesicles. Here, we used cryo electron tomography to analyze the three-dimensional architecture of HSV1 in axons of hippocampal neurons. In mid-axonal regions we found predominantly non-enveloped capsids. Interestingly, we observed both genome-containing and empty capsids that differed significantly in the amount of bound proteins. Viral protein recruitment thus varied between the different cytosolic capsid types, but effective transport occurred despite these differences. Furthermore, we observed three-dimensional snapshots of secondary capsid envelopment in axon terminals. Altogether, this study provides valuable structural detail on axonal HSV1 particles supporting the notion that viral subassemblies are conveyed along the axons to be assembled only after axonal transport.

## Introduction

Herpes simplex virus type 1 (HSV1) is the prototype of the *Alphaherpesvirinae,* a subfamily of the *Herpesviridae*. Viruses of this subfamily establish lifelong latent infections in the nervous system of the host organism. About 80 percent of the human population is infected with HSV1. The infection is typically manifested by cold sores near the oral cavity but can also provoke ocular lesions and in rare cases encephalitis. The pleomorphic HSV1 virion has a complex structure [1], [2]: the viral capsid encloses the double-stranded DNA genome and is surrounded by an amorphous layer of more than 20 different tegument proteins. A membrane envelope with inserted glycoproteins forms the outer boundary of the viral particle. The capsid is an icosahedrally symmetric protein shell with a diameter of 125 nm and composed of 162 capsomers [3]–[7]. Of these capsomers, 150 are hexons, 11 are pentons and one is the portal [8]–[10], responsible for DNA packaging into the capsid after procapsid assembly. The pentons and the portal are located at the 12 vertices. The structures connecting adjacent capsomers are termed triplexes.

HSV1 capsids assemble in the nucleus with the help of a scaffold protein [11]. The nuclear capsids have been classified into four types: round procapsids and angular A-, B- and C-capsids. Both A- and B-capsids are devoid of DNA, but B-capsids retain the scaffolding protein [12]. C-capsids are mature capsids containing the viral genome [11]. C-capsids have been reported to contain larger amounts of the minor capsid proteins pUL17 and pUL25 than A- and B-capsids [13]–[16]. When compared to virions, nuclear capsids are virtually devoid of tegument [16]–[20], although it has been suggested that association of the tegument proteins pUL36 and pUL37 might occur already in the nucleus [21], [22]. Primary envelopment of the newly assembled capsids takes place at the inner nuclear membrane (reviewed in [19]). It is followed by fusion of the primary envelope with the outer nuclear membrane leading to de-envelopment of capsids. Once released into the cytosol, progeny capsids are transported towards the site of secondary envelopment [19], [23]–[25].

In neurons, egressing HSV1 particles are transported over long distances. For alphaherpesviruses the nature of the particles undergoing axonal transport from the nucleus to the cell periphery (termed anterograde transport) is debated, in particular for HSV1 and pseudorabies virus (PrV). Two models of viral assembly have been suggested. According to the ‘separate model’ (synonym: subassembly model) [24], [26], [27], cytosolic capsids lacking an envelope are transported along microtubules while the viral tegument proteins and glycoproteins travel separately or in association with transport vesicles. In this model, assembly of mature virions occurs at axonal varicosities or at axon terminals [17], [26]–[32]. Conversely, according to the ‘married model’, viral particles are transported from the cell body towards the axon terminal already fully assembled and inside transport vesicles [33]–[37]. In both models, the HSV1 particles are transported in the cytoplasm along microtubules by cellular microtubule motors (reviewed in [24], [38]–[40]). One particular feature of all cytoskeletal motors is that they move actively only in one direction: either to the plus-ends of microtubules close to the plasma membrane, or towards the minus-ends that are clustered at the microtubule-organizing center situated in close proximity to the cells nucleus. Recent biochemical data have provided evidence that tegumented capsids can recruit several copies of microtubule motors of opposing polarity simultaneously [16]. Furthermore, the intracellular movement of individual particles occurs in both directions with occasional changes in direction; nevertheless, there is an overall preferred transport direction for all viral and host cargos [41], [42]. The microtubule motor dynein is responsible for transport of capsids from the cell periphery towards the nucleus (termed retrograde transport) [43], [44]. For this function, dynein requires the interaction with its cofactor dynactin [45]–[47]. Anterograde transport, *i.e.* transport in the opposite direction, is mediated by plus-end-directed microtubule motors, such as kinesin-1 or kinesin-2 [16], [39], [43], [44]. Several tegument proteins are essential for intracellular transport of capsids and may contribute to forming viral motor binding sites. In particular, it has been shown that the tegument proteins pUL36 and pUL37 are essential for capsid transport during entry and egress [48]–[50]. Moreover, HSV1-GFPVP26 capsids lacking most of the outer tegument proteins, but still containing inner tegument proteins such as pUL36 and pUL37, are transported along microtubules *in vitro* in the presence of cytosol [18]. Furthermore, Radtke *et al.* [16] have shown that pUL36 and pUL37 are accessible on the surface of capsids that recruit motors *in vitro*. It has also been suggested that the capsid protein VP26 is involved in retrograde transport of capsids [51], although other studies have demonstrated that this protein is not essential for dynein-mediated transport [52]–[54]. Furthermore, VP26 is also not required for recruiting dynein and kinesin onto isolated capsids *in vitro* [16], [18]. The tegument protein pUS11 was shown to bind to kinesin-1 [55], although it does not appear necessary to recruit kinesin-1 to capsids *in vitro* [16].

Little is known about the location and identity of the tegument proteins bound to capsids *in situ* during transport. A previous study of HSV1 virions using cryo electron microscopy and single particle icosahedral reconstruction has revealed only a small ordered density of tegument located at the vertices of the capsid [56]. This density has been suggested to be formed by the inner tegument protein pUL36. Furthermore, earlier conventional electron microscopy studies have shown capsids inside cells with substantial densities bound at the vertices [45]. Together, these results have suggested that the molecular motor complexes might attach to the vertices of the capsid but this binding platform has remained uncharacterized.

Here, we applied cryo electron tomography (cryoET, [57]) to analyze the three-dimensional structure of HSV1 particles during anterograde axonal transport. By virtue of this technique, the rapidly frozen specimen is kept vitrified in near-native conditions [58], [59] and does not suffer from structural re-arrangements caused by chemical fixation, dehydration or heavy metal staining. The vast majority of progeny capsids found in axons were non-enveloped; hence, our data support the separate model of anterograde axonal transport. Surprisingly, not only cytosolic capsids containing DNA but also capsids devoid of DNA had been transported along the axons despite significant differences in the amount of tegument associated with them. Further, three-dimensional cryoET snapshots of capsid assembly by secondary envelopment in axon terminals suggest that secondary envelopment might involve vesicle fusion to form a sufficiently large enveloping compartment.

## Results

### Establishing an experimental system of viral intra-axonal transport accessible to cryoET

Intracellular transport of alphaherpesviruses is often studied in neurons of dissected nervous ganglia explants, *e.g.* in sympathetic neurons of rat superior cervical ganglia (SCG). Unfortunately, this system is not accessible to analysis by cryoET for two reasons. First, the size of the explants is typically prohibitive as, upon freezing, it causes an ice thickness exceeding the penetration limit of electrons (∼1 µm) [57]. Placing the EM grids further away from the explant to analyze flat regions of the outgrown neurons is likewise not applicable because the grids then needed to be removed prior to freezing. In this case, the neurons would be damaged thus preventing a native *in situ* analysis. Therefore, we chose to use primary neurons cultured after dissociation.

In early experiments, we analyzed primary neurons from dissociated rat dorsal root ganglia (DRG) by cryoET. These peripheral sensory neurons present a near-native model for studying HSV1 transport. Unfortunately, also in this system, the prominent thickness of the cell bodies resulted in a specimen thickness that impeded cryoET. The hippocampus is a brain region that is typically infected during herpes simplex encephalitis in humans [60]. Therefore, we next analyzed hippocampal neurons, which are also a close-to native system for the study of HSV1 infection and, in addition, provided extended areas suitable for cryoET of axons.

### Fluorescence microscopy imaging of progeny HSV1 capsids in axons of primary neurons

**Figure 1:**
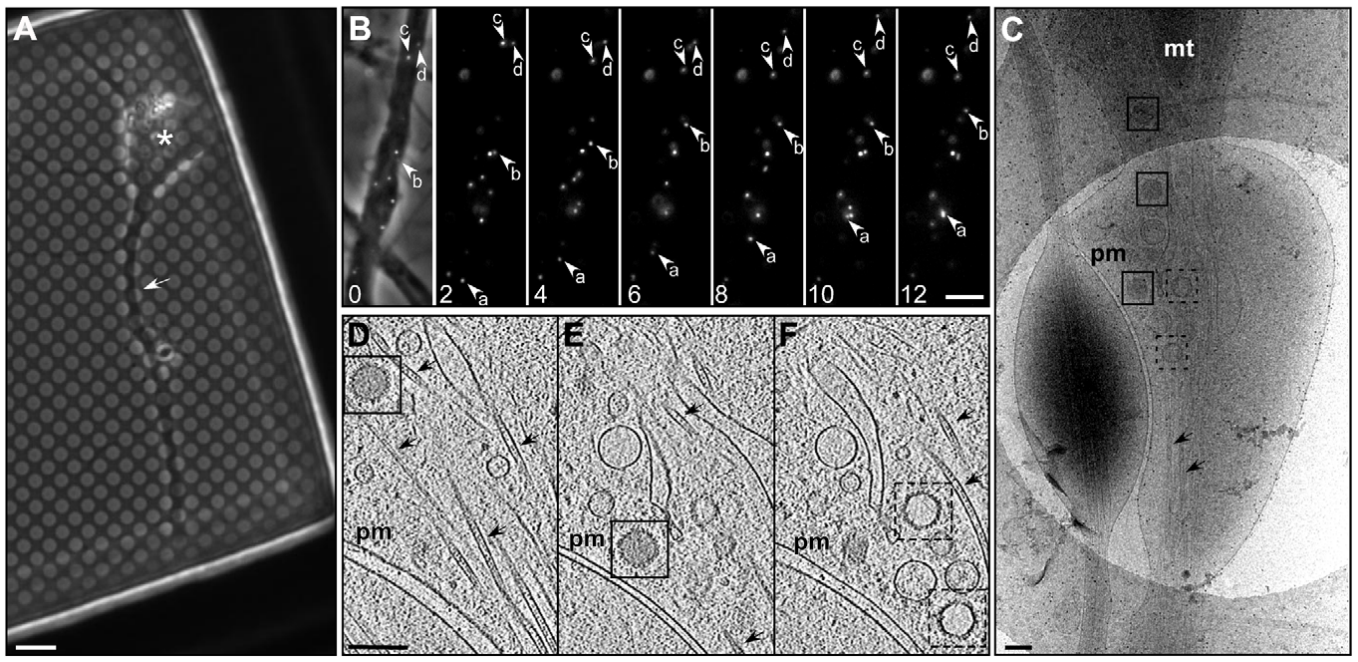
Transport of HSV1 capsids in neurons during egress. (A) Bright field image of a hippocampal neuron grown on a holey carbon support film for 7 days. A neurite (white arrow) and cell body (asterisk) are indicated. Bar: 6 µm. (B) Series of time-lapse wide-field fluorescence images of a mid-axon region of a HSV1(KOS)-GFPVP26 infected neuron at 16 hours p.i.. Pictures were taken every 2 seconds, as indicated by the number in the lower left corner. The left image shows an overlay of the fluorescence channel with the bright field image. Arrows indicate the positions of individual viral particles. Bar: 5 µm. (C) Cryo electron microscopy (projection image) of an intact axon at 16 h p.i.. Cytosolic C-capsids are framed in black and cytosolic A-capsids in black dashed squares. (D–F) Slices through the reconstructed tomographic volume obtained from the area of interest in (C). pm: plasma membrane; mt: mitochondria; black arrows: microtubules. Bars in (C–F): 200 nm.

**Figure 2:**
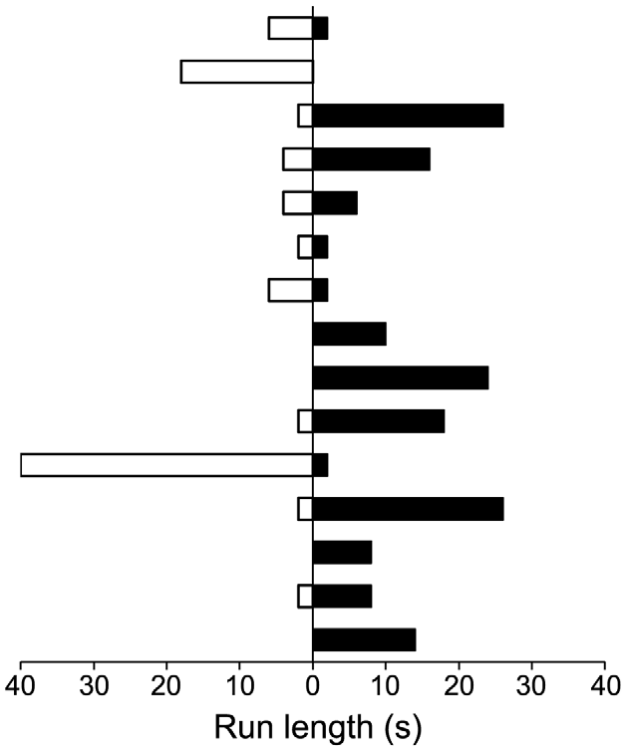
Directionality and run lengths of intracellular transported viral particles at 16 h p.i.. Each bar corresponds to an individual single run, 15 in total. Particles that entered or ran out from the field of view during the 10 min observation period were also taken into account. These data are coming from two different observations. For more detail see Table S1. Black bars: anterograde transport, white bars: retrograde transport.

Primary hippocampal neurons cultured on electron microscopy grids (Figure 1A) were infected with HSV1. Infection was followed by live cell imaging using HSV1(KOS)-GFPVP26 [52]. VP26 is a small capsid protein located on top of the hexons [61], [62]. At 2 h post infection, the axons contained only occasionally fluorescent HSV1 particles (not shown). Around 16 hours post infection (p.i.), after synthesis of progeny viruses, massive egress of fluorescent viral particles occurred (Figure 1B). Around 60% of the fluorescently labeled particles were motile. The average velocity of the viral particles during anterograde transport was 2.4 µm/s (Figure 2, Table S1), consistent with earlier reports [28], [42], [48], [49]. Despite frequent changes in direction, capsid transport along axons had a preferred orientation towards the cell periphery and anterograde transport on average was faster than retrograde transport (Figure 2, Table S1).

### Cytosolic non-enveloped progeny capsids were transported in axons

Samples vitrified at 16 h p.i. were analyzed by cryo electron microscopy. Often, at least two capsids per field of view (1.65×1.65 µm) were recognized by cryo electron microscopy (cryoEM) 2D projection images (Figure 1C). Using cryoET, we identified the transported particles as non-enveloped cytosolic capsids (Figure 1D–F). In the axon, they were consistently found in close proximity to microtubules (Figure 1D–F; arrows).

### Axon cytosol contains not only C-capsids but also A- and B-capsids

Besides cytosolic DNA-containing C-capsids, unexpectedly, there were also cytosolic A- and B-capsids present in the axons, although in lower numbers than C-capsids. In 2D projections of thicker cellular specimens, structural features were superimposed upon one another, and thus difficult to interpret. In contrast, in three-dimensional tomographic reconstructions, the capsids were clearly discernible from cytoplasmic vesicles and finer features such as individual capsomers as well as the structural elements in the cytoplasm became recognizable (Figure 1D-F). We could clearly identify cytosolic A-capsids as well as cytosolic B-capsids. While the former were angular and empty, the latter contained densities of the scaffold protein in the capsid lumen. Their morphology was clearly different from that of fully DNA-packaged C-capsids (Figs. 1D–F, S1). We noted that cytosolic A- and B-capsids were not observed after infection with HSV1(KOS) or the HSV1(KOS)-GFPVP26 variant , whereas they comprise the majority in HSV1(F) (Table 1). The microtubules had luminal densities consistent with earlier observations [63] (Figure 1D–F). While dense material was associated occasionally with cytosolic capsids (data not shown), we could not assign unequivocally such densities to cellular microtubule motors. Overall, the capsids seem to contain very little tegument, and the capsomers were not obscured by tegument or cellular protein complexes but were clearly recognizable.

**Table 1:** Frequency of viral particle types found in middle regions of axons for the HSV1 strains used for infection.

HSV-1 strain	Cytosolic A-capsids	Cytosolic B-capsids	Cytosolic C-capsids	Enveloped virions	Number of tomograms
**F**	6	20	13	3	7
**KOS**	0	0	4	3	5
**KOS-GFPVP26**	0	0	24	0	2

### Icosahedral vertices of axonal capsids are the sites of capsid-tegument interaction

**Figure 3:**
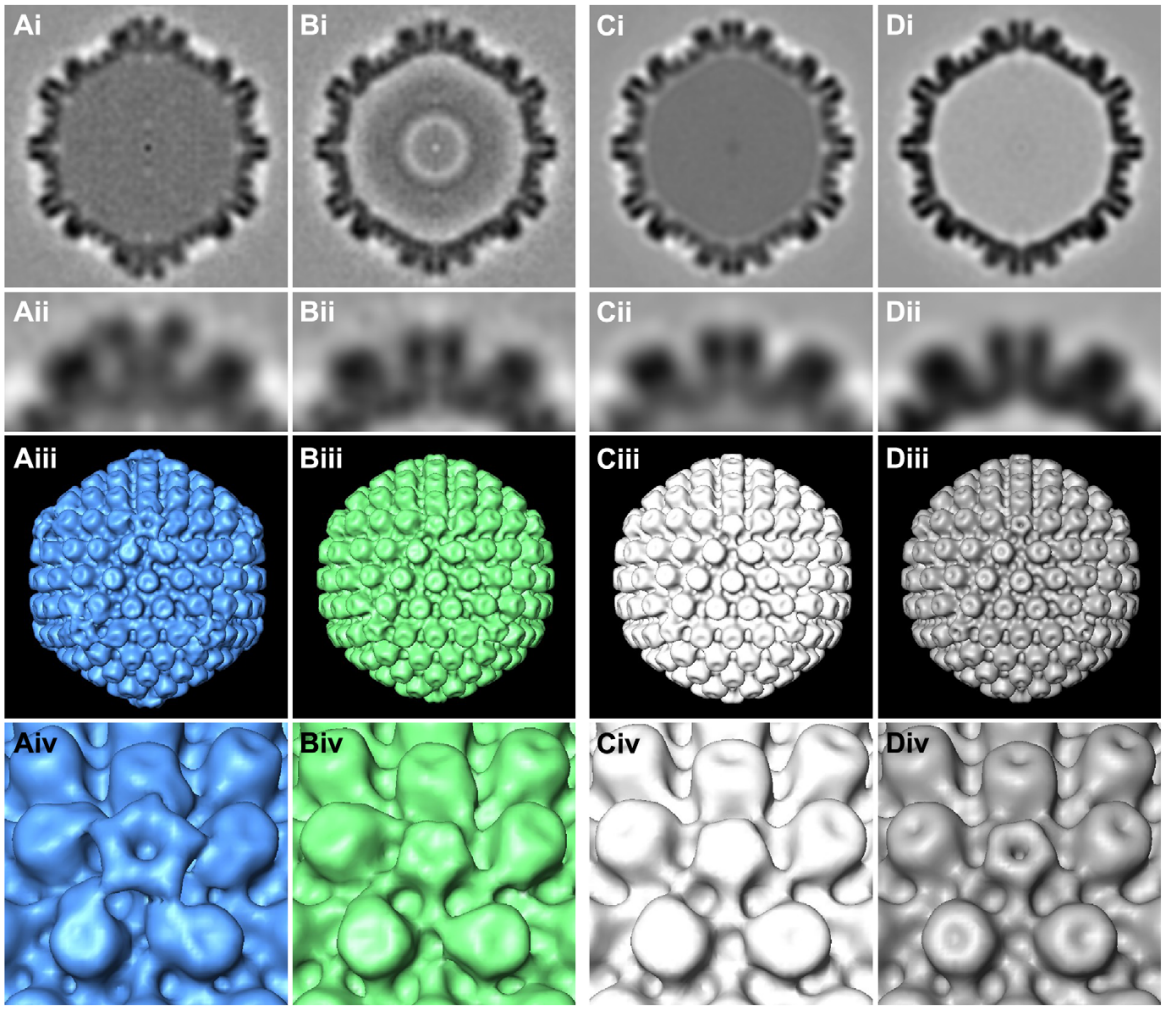
Subvolume averaging of cytosolic capsids. Results from subtomogram averaging are presented for (Ai-iv) 41 cytosolic C-capsids, (Bi-iv) 26 cytosolic A-/B-capsids, (Ci-iv) 143 nuclear C-capsids and (Di-iv) 158 nuclear A-capsids. Row (i): central cross sections of the averages. Row (ii): close-up view of the top vertex in row (i). Row (iii): isosurface representation of the averages with a threshold of 1.5σ above the mean density. Row (iv): close-up view of a vertex from row (iii).

**Figure 4:**
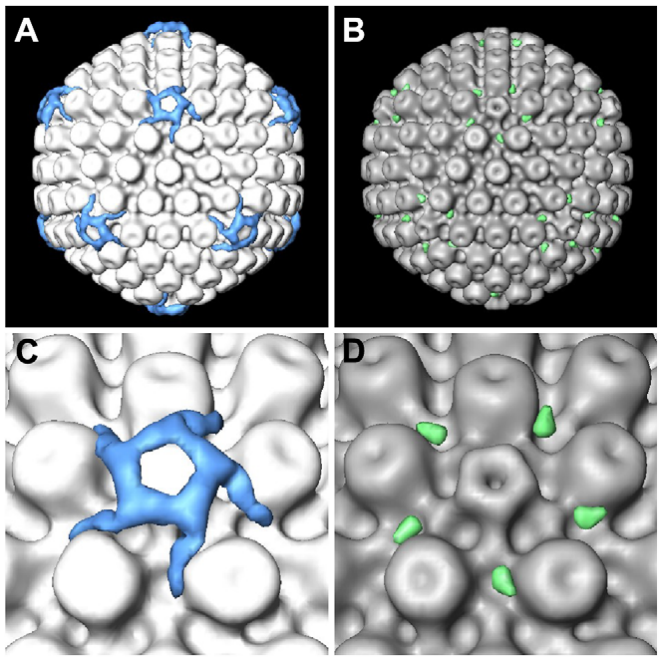
Difference maps between cytosolic capsids and nuclear capsids. (A) Difference map between cytosolic C-capsids and nuclear C-capsids, superimposed onto the nuclear C-capsids average. (B) Difference map between cytosolic A-/B-capsids and nuclear A-capsids, superimposed onto the nuclear A-capsids average. (C, D) Close-up view of a vertex in (A) and (B), respectively. The isosurface thresholds for the difference maps are 1.5σ above the mean density in (A) and 0.5σ in (B).

Earlier studies have shown that some tegument proteins are essential during intracellular capsid transport [48]–[50], [64], [65]. To analyze the interactions between capsid and tegument during transport, we averaged the densities of cytosolic capsids that were computationally extracted from tomograms of infected neurons. The 14 tomograms acquired of neurons infected either with wild type strains HSV1(F) or HSV1(KOS), or with HSV1(KOS)-GFPVP26 contained a total of 67 cytosolic capsids (Table 1). We calculated separate capsid averages for 41 cytosolic DNA-containing C-capsids (24 HSV1(KOS)-GFPVP26, 4 HSV1(KOS) and 13 HSV1(F) ; Table 1) (Figure 3Ai-iv) and for 26 cytosolic DNA-lacking A- or B-capsids (6 A-capsids and 20 B-capsids, all HSV1(F) wild-type, Table 1) (Figure 3Bi-iv). The resolution of the averages was 6.9 nm and 9.7 nm, respectively. Cytosolic C-capsids were compared to the average of 143 nuclear C-capsids, *i.e.* C-capsids biochemically purified from the nuclei of infected cells (Figure 3Ci-iv; Figure 4A, C). Cytosolic A-/B-capsids were compared to the average of 158 nuclear A-capsids, likewise biochemically purified from nuclei of infected cells (Figure 3Di-iv; Figure 4B, D). The resolution for both groups of nuclear capsid averages was 5.6 nm. Nuclear capsids are known to be virtually devoid of tegument proteins [16]–[19]. Therefore, a comparison of native cytosolic capsids to biochemically purified nuclear capsids could reveal features on cytosolic capsids that correspond to tegument proteins acquired shortly before nuclear egress or in the cytosol. Indeed, this comparison revealed a prominent extra density, located exclusively at the C-capsid vertices (Figure 4A, C; blue). It was present on top of the pentons and connected further to the positions of the two adjacent triplexes and to one side of the neighboring hexons. Thus, these extra densities were only positioned on hexon-penton interfaces but not on hexon-hexon interfaces.

### Cytosolic A-/B-capsids possess less tegument density at the capsid vertices than cytosolic C-capsids

The comparison of cytosolic A-/B-capsids to the nuclear A-capsids showed that cytosolic A-/B-capsids comprised only a small amount of extra density (Figure 4B, D; green). Nevertheless, this extra density was also located exclusively at the vertices, in particular towards one side of the peripentonal hexons. On cytosolic A-/B-capsids, no extra density was present on top of the pentons.

### Few enveloped virions in middle regions of axons

**Figure 5:**
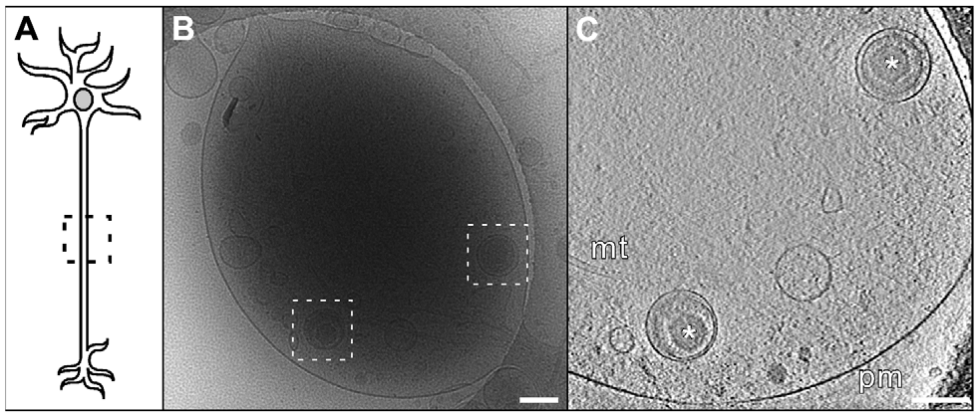
Enveloped virions in middle regions of axons. (A) Schematic diagram of a neuron indicating the mid-axon region. (B) Cryo electron microscopy (projection image) of a pair of enveloped virions (boxed areas) in a mid-axon region vitrified at 16 h p.i.. Note the bundle of microtubules entering and leaving this area. Bar: 200 nm. (C) CryoET slice through the respective reconstructed tomographic volume for the field shown in (B). Asterisk: enveloped capsids; mt: microtubule; pm: plasma membrane. Bar: 200 nm.

Occasionally, there were enveloped virions in regions of the axons that were quite some distance away from both, the soma and the axon terminals (Figure 5), although at much lower frequency than non-enveloped capsids. Of the 73 capsids located in middle regions of axons at 16 h p.i., less than 10% were enveloped while the others were non-enveloped (ratio 6∶67). Nevertheless, such enveloped virions were also located in close proximity to microtubules (data not shown).

### Secondary envelopment occurs in axon terminals

**Figure 6:**
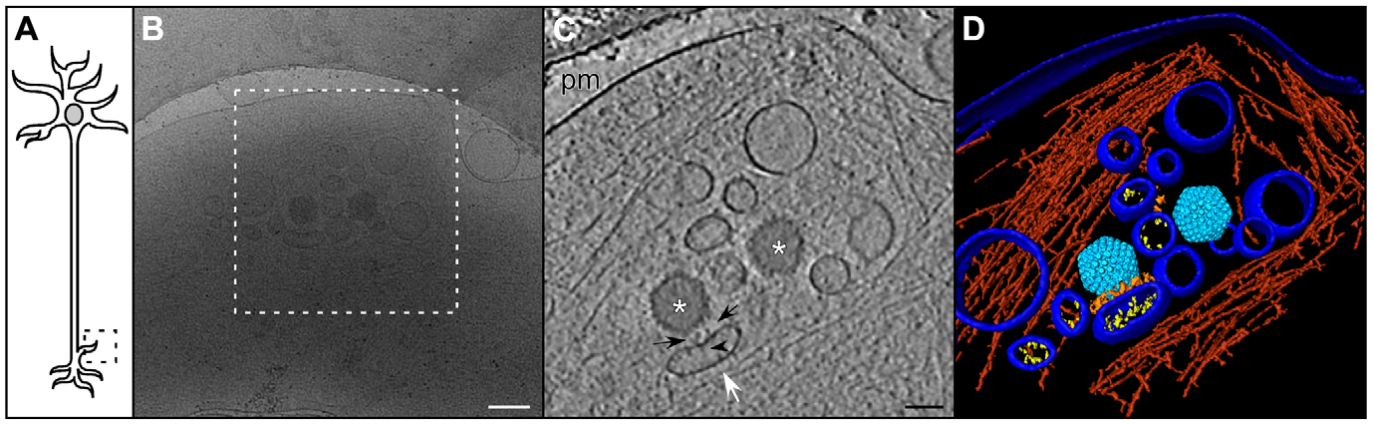
Secondary envelopment at axon terminals. (A) Diagram of a neuron indicating an axon terminal. (B) Cryo electron microscopy (projection image) of an intact axon terminal at 16 h p.i.. Bar: 200 nm. (C) Slice of the tomogram taken in the area highlighted in (B), showing secondary envelopment of capsids. Asterisks: capsids; white arrow: enveloping vesicle; arrow head: glycoproteins; black arrows: tegument; pm: plasma membrane. Bar: 100 nm. (D) Surface rendering of the tomogram shown in (C). Capsid (light blue), tegument (orange), glycoproteins (yellow), actin (red), plasma membrane and vesicle membrane (blue).

Secondary envelopment sites were characterized by capsids being in close proximity to groups of vesicles (Figure 6). Three-dimensional analysis revealed that such assembly sites lacked any cytoskeletal elements, but that filamentous actin rather surrounded these assembly sites (Figure 6C, D). In contrast, there were no microtubules in these areas. Notably, the vesicles in assembly sites had different sizes and were characterized by two different morphologies. Some of the vesicles were studded with spike-like densities, protruding from the membrane into the lumen of the vesicle (Figure 6C, black arrowhead, Figure 6D, yellow densities). In contrast, other vesicles showed a smooth luminal surface. Typically, electron-dense material, presumably tegument, was accreted on the cytoplasmic face of vesicles with spike-like densities on their interior side (Figure 6C, black arrows). When those vesicles had a concave cytoplasmic side it was typically facing towards a capsid. By virtue of our three-dimensional reconstructions, we revealed that at least in some cases the volume of individual spike-studded vesicles appeared not to suffice to fully enclose a capsid.

## Discussion

In this study, we used cryoET to visualize HSV1 capsid-tegument interactions in 3D during axonal transport in vitrified hippocampal axons. To this end, we first established a close-to-native experimental cell system enabling us to follow intra-axonal herpesvirus transport by both fluorescence microscopy and cryoET. By culturing dissociated hippocampal neurons directly on electron microscopy grids, we were able to circumvent the practical limitations that dissected nerve ganglia explant systems pose for cryoET. Furthermore, this type of primary neurons provides a relevant model for HSV1 since the hippocampus in the brain is infected during herpes simplex encephalitis in humans [60].

Culturing the hippocampal neurons on electron microscopy grids did not impair the course of HSV1-infection. Live-cell imaging using HSV1(KOS)-GFPVP26 revealed a peak in anterogradely transported virus particles at 16h p.i.. This time point is consistent with earlier reports on egressing HSV1 in infected neurons [29], [36], [66], [67]. The average anterograde speed of 2.4 µm/s of the HSV1 particles (Figure 1C, Table S1) agrees well with earlier observations [41]. These transport rates suggest active transport by a kinesin, *e.g.* kinesin-1 or kinesin-2 which have been shown to bind to isolated, tegumented HSV1 capsids *in vitro* [16]. The characteristic pattern of net anterograde transport despite intermittent changes in directionality has likewise been reported before for herpesvirus egress [41], [42]. It most likely reflects the engagement of motor complexes with opposite directionality on the same viral particle [16], [68]. A direct correlation between the live-cell fluorescence imaging and cryoET at the level of individual viral particles was impossible since the handling steps between fluorescence imaging and the time point of vitrification took about 20 to 30 s. CryoET is not the adequate tool for a systematic, statistical analysis, since it is limited to axon areas thin enough to be penetrated by the electron beam. Nevertheless, on a population level, the number of egressing HSV1 particles was fully sufficient to characterize particles in transit at higher resolution by cryoET. The majority of the viral particles in mid-axon regions were non-enveloped cytosolic capsids.

We next focused on the organization of tegument proteins on these cytosolic, axonal capsids to identify the interaction platform for the attachment of microtubule motors mediating intracellular transport. For HSV1(F), we detected all three intra-axonal capsid types – 52% contained DNA (cytosolic C-capsids), while the remaining ones lacked DNA (15% cytosolic A- and 33% B-capsids, respectively). We also frequently observed A- and B-type capsids during and after secondary envelopment in axon terminals (data not shown). In contrast to a prevailing hypothesis [13], [69], our results indicate that A-/B-capsids leave the nucleus and are actively transported to the cell periphery as it had been suggested previously [3], [70]. Cytosolic B-capsids have also been reported for another herpesvirus, simian cytomegalovirus [71]. Whether the fact that the majority of intra-axonal capsids were cytosolic C-capsids reflects their higher efficiency in nuclear egress or just the ratio of nuclear C-capsids to A-/B-capsids remains to be determined. Formally, we cannot exclude that the packaged DNA genome had been lost at a later stage in the cytosol resulting in the appearance of cytosolic A-type capsids. However, it seems very unlikely that cytosolic C-capsids would give rise to cytosolic B-capsids.

Interestingly, we observed cytosolic A-/B-capsids only when infecting with HSV1(F) , while neurons infected with HSV1(KOS) (wild-type or -GFPVP26) lacked them (Table 1). Negatsch *et al.* reported recently for HSV1(KOS) a lack of pUS9 expression [72]. The US9 region of our HSV1(KOS) has the same mutations as reported by Negatsch *et al.* (2011), while our HSV1(F) lacks any mutations in the pUS9 region (data not shown). pUS9 had earlier been reported to play a role in herpesvirus axonal transport [32], [73]. Our results suggest that also nuclear egress of the HSV1(KOS) capsids may be either highly specific for C-capsids or impaired for A-/B-capsids when compared to HSV1(F). Whether this difference due to the changes in the US9 gene remains to be established. Thus, in a situation where nuclear capsid egress is highly specific for C-capsids as observed here for HSV1(KOS), A- and B-capsids might be retarded in the nucleus.

The late time point of vitrification at 16 h p.i. suggests that both cytosolic, axonal progeny C-capsids and A-/B-capsids presented an adequate tegument composition to be transported towards the cell periphery for assembly and exit. The abundance of potential parental, incoming capsids derived from newly produced viruses superinfecting these axons is very low, because there were virtually no capsids in cells that had been vitrified 5 to 20 minutes p.i. even when using an MOI of up to 200 (data not shown).

The combination of cellular cryoET with subvolume-averaging allowed visualizing the tegument density distribution on intra-axonal capsids in unprecedented detail. Subvolume-averaging for macromolecules inside cells has been barely performed so far [74]. The reasons for this are that it is difficult to identify macromolecular complexes within a cellular context, and that the number of complexes of interest within cells is low compared to *in vitro* particle preparations. Comprehensive knowledge on the protein composition of the intra-axonal cytosolic capsids is lacking since so far they could not be purified for quantitative mass spectrometry analysis. The existing information on these particles is based on data using fluorescently tagged proteins and immunolabelling experiments and therefore incomplete. Here, we obtained novel information by averaging subviral structures in their native surroundings. This will enable future studies on their interactions with other host factors, and will allow to correlate such data derived from *in situ* / *in vivo* experiments with the results of biochemical systems reconstituting key intermediate steps *in vitro* [75].

In our study, subvolume-averaging *in situ* including icosahedral symmetrisation showed that tegument proteins associated exclusively with the capsid vertices. In accordance with previous studies from isolated virions [61], the capsid protein VP26 did not contact the extra density present on such cytosolic capsids. This supports the notion that VP26, located on top of the capsid hexons [6], but not the vertex pentons, is dispensable for capsid transport [51]-[54]. Our results furthermore agree with biochemical studies that VP26 is not required for recruiting dynein or kinesin-1 onto capsids [16], [18]. In turn, the tegument material exclusively located around the capsid vertices supports the notion that the molecular motors mediating transport might bind to the vertex region as it has been suggested previously [45].

The striking differences in the tegument structure between cytosolic C-capsids and cytosolic A-/B-capsids (Figs. 3 and 4), most notably the presence of extra density on top of the pentons of the cytosolic C-capsids, provided valuable insights into the complex capsid–tegument interaction network. The minor capsid protein pUL25 forming a heterodimer with the protein pUL17 has been attributed to a density termed “elongated C-capsid specific component (CCSC)” in cryoEM reconstructions of nuclear C-capsids [13], [76]. The CCSC is barely visible on nuclear A- and B-capsids, consistent with a lower abundance of both proteins that has also been confirmed by proteomic data [13], [77]. Both pUL25 and pUL17 have been localized on nuclear A- and B-capsids by cryoEM and TAP pulldown assays [76], [78]. In these studies the protein complex has been termed “capsid-vertex specific component (CVSC). Furthermore, pUL25 can interact with pUL36, the largest herpesvirus tegument protein, in HSV1 [50], and in PrV [22] that in turn interacts with the tegument proteins pUL37 and VP16 [65], [80]–[82]. In accordance with these studies, our results show that cytosolic C-capsids were associated with a higher amount of tegument than cytosolic A-/B-capsids. This is consistent with the cytosolic C-capsids comprising higher amounts of pUL17/pUL25, and therefore binding more tegument than cytosolic A-/B-capsids. Nevertheless, this low tegumentation on cytosolic A-/B-capsids appeared to be sufficient for at least some capsid transport from the neuronal soma into the axons. Thus, full coverage of all capsid vertices by tegument seems not to be required for microtubule transport, and tegument recruitment onto even one vertex might be sufficient albeit barely detectable in the icosahedral average reconstruction of cytosolic A-/B-capsids.

Non-enveloped HSV1 cytosolic capsids detected inside hippocampal axons are in agreement with the ‘separate model’ of alphaherpesvirus axonal anterograde transport [17], [24], [26]-[31]. Further supporting this model, we identified sites of secondary envelopment at axon terminals (Figure 6). We also observed enveloped virions in mid-axon regions (Figure 5), but at a much lower rate than non-enveloped particles (Table 1). CryoET is not an adequate tool for a statistical analysis, but the ratios between enveloped and non-enveloped capsids nevertheless indicate a trend. Two different scenarios may explain this. First, it is possible that the virions in middle regions of axons underwent secondary envelopment in a varicosity, and that they would eventually exit the cell also from here as reported previously [29]. This would imply that even though enveloped viral particles were sporadically observed, they might not undergo long distance transport. Although axon terminals appeared as the main envelopment and exit sites for HSV1, some enveloped particles may have been generated in the soma, and only afterwards entered the axons. A recent report comparing PRV and several strains of HSV1 reports that in explanted primary neurons from rat superior cervical ganglia, for HSV, about 75% of the viral particles in the axon and growth cone were enveloped and 25% non-enveloped [67]. Thus, the assembly pathway of HSV1 may be more complex than anticipated by the ‘married’ or ‘separate’ models for HSV1 axonal transport. Further studies comparing a wider range of neurons derived from different structures of the nervous system and other strains of alphaherpesviruses will ultimately reconcile these apparent discrepancies. Furthermore, combinations of different tags on VP26 with additional mutations in US9 or other herpesviral genes may result in complex phenotypes in axonal transport that may not be recognized or remain silent during infection of epithelial cells.

CryoET is the method of choice for visualizing filamentous actin. Our native three-dimensional analysis of the axon terminals revealed that the secondary envelopment sites themselves were devoid of filamentous actin while the actin meshwork surrounding them seemed to form the boundary of an assembly compartment. Given that the dimension of these compartments was around (1 µm)^3^, these surrounding actin filament structures remained nevertheless unnoticed by fluorescence microscopy so far. Future dedicated studies of such actin cages using correlative fluorescence and electron microscopy are needed to further characterize this feature of assembly sites.

The assembly sites contained numerous vesicles studded on their luminal inside with glycoprotein-like densities, presumably having being transported to these sites independently of cytosolic capsids. Classical electron microscopy techniques cannot visualize these spikes as unequivocally as it has been achieved here. Tegument proteins accumulated on the cytosolic surface of these vesicles and might be the cause of a vesicle indentation to form a concave surface towards the capsids. Further, some of these vesicles did not appear to be large enough to fully envelope one capsid. The close proximity of several of these vesicles suggests that secondary envelopment might involve vesicle fusion to form a sufficiently large enveloping compartment.

In summary, we have characterized a new neuronal infection model that enables investigating axonal transport, assembly and egress of HSV1 in 3D in a close-to-native state. CryoET revealed that the axonal viral particles were predominantly non-enveloped cytosolic capsids. We found that in addition to cytosolic C-capsids, unexpectedly cytosolic A-/B-capsids also underwent axonal transport. The prominent differences in tegumentation between these two capsid types suggest that efficient transport of capsids does not require large amounts of tegument, and occurs in the presence of different amounts of tegument. For both capsid types, the capsid-to-inner-tegument interactions were exclusively limited to the capsid vertices. These interactions are likely crucial for transport by forming a binding platform for microtubule motors. The higher abundance of non-enveloped over enveloped capsids in middle regions of axons, and the secondary envelopment sites at axon terminals favor the separate model for HSV egress for this combination of HSV1 strains and hippocampal neurons. The three-dimensional visualization of secondary envelopment sites revealed insights into a level of detail that allowed us to propose novel aspects of this process like formation of an actin bound compartment and a possible role for fusion of smaller vesicles during envelopment.

## Materials and Methods

### Viral preparation

HSV1(F), HSV1(KOS) and HSV1(KOS)-GFPVP26 [52] virions were amplified in BHK-21 cells, and the viral titers were determined by plaque titration on Vero cells as described previously [1], [54]. The virus stocks had a titer of 10^9^ PFU /ml.

### Fluorescence microscopy and time-lapse analysis

Hippocampal neurons were isolated from 17 days old rat embryos (provided by Boyan Garvalov, MPI Neurobiology, Germany). IBIDI slides (µ-slide 8 well, Ibidi GmbH) were coated with 1 mg/ml poly-L-lysine (Sigma) in borate buffer (1.24 g boric acid + 1.9 g borax in 400 ml distilled water, pH 8.5) overnight. They were then washed with distilled water three times before adding MEM horse serum medium (Gibco), which was replaced next day by neurobasal medium (Gibco) supplemented with B27 (Gibco) and glutamine. Neurons were then seeded at a density of 4,500 cells per 1 cm^2^. They were incubated for 7 days at 37°C, 5% CO_2_ and then infected with HSV1(KOS)-GFPVP26 at an MOI of 50 PFU/cell. Infected neurons were imaged using a 63x oil objective on an Axiovert 200 M light microscope (Zeiss) equipped with an AxioCam HRm camera (Zeiss) and controlled by the Axiovision 4.1 software (Zeiss). For the long time-lapse experiments, viral infection of neurons was monitored by wide field phase contrast and fluorescence imaging every hour over a period of 24 hours. For the short time-lapse experiments, fluorescent pictures were taken every 2 seconds at the same region for 10 min. Fluorescence was detected with a GFP blue band excitation/green band emission filter set (HQ-EGFP; F41-017; AHF Analysentechnik AG). An incubation chamber around the microscope allowed time-lapse observations at 37°C, 5% CO_2_ and high humidity (EMBL workshop; No. 530010; Cell Biology Trading). In the short time-lapse experiments, the speed and length of several continuous runs were measured in two different observations from two different neurons (Table SI). Some of the particles came in or moved out of the field of view during the observation time. These were also taken in consideration in the measurements.

### Infection of neurons on grids

Au grids of 200 mesh with holey carbon support films (Quantifoil GmbH, Jena, Germany) were sterilized on a Petri dish under UV light for 15 min and then coated as described above for the IBIDI slides. Dissociated rat hippocampal neurons were prepared as described [83]. Neurons were plated over the grids at a density of 100,000 cells in a 60 mm diameter Petri dish and incubated 7 days at 37°C and 5% CO_2_ to enable the growth of axons and dendrites. Neurons were then infected with HSV1 at a MOI of 50. At 16 hours post infection (p.i.), neurons were prepared for cryoET as described below.

### Purification of nucleocapsids

BHK-21 cells were infected with 0.01 to 0.02 PFU/cell for 2 to 3 days until detached and collected by sedimentation. They were then washed once in MNT buffer (30 mM MES, 20 mM Tris, pH 7.4, 100 mM NaCl), snap-frozen and stored at -80°C. Nuclear capsids were purified as previously described [16], [18], [21], [84]. Capsids were diluted in three volumes TNE (20 mM Tris, pH 7.5, 500 mM NaCl, 1 mM EDTA) with 10 mM DTT and protease inhibitors, and sedimented by centrifugation (Beckman TLA100.2 rotor, 15 min, 50 krpm, 4°C). The pellets were resuspended in BRB80 buffer (80 mM PIPES, 1 mM EGTA, 2 mM MgCl_2_; pH 6,8 with KOH) with 10 mM DTT, 1 mg/ml soybean trypsin inhibitor, protease inhibitors, 100 mg/ml RNase (Roth, Germany) and 0.1 U/ml DNase I (M6101, Promega, USA)). Capsids were then incubated for 30 min at 37°C and overnight at 4°C, repelleted (TLA100.2 rotor, 8 min, 50 krpm, 4°C) and resuspended in BRB80 buffer by tip sonification on ice (3×10 seconds, 40 W).

### Cryo electron tomography

Hippocampal neurons growing adherently on the holey carbon support film on Au grids were prepared for cryoET as follows: 2 µl of colloidal gold suspension (10 nm diameter in HBSS buffer, coated with BSA) was added on the EM grid. Excess of liquid was removed by blotting the grid with a filter paper. Specimens were vitrified by plunge-freezing into liquid ethane and transferred into liquid nitrogen for storage. In the case of isolated capsids (salt-treated capsids and nuclear capsids), 5 µl droplets were added onto the holey carbon support film on Cu 200 mesh Quantifoil grids. To avoid formation of aggregates, salt-treated capsids were sonicated before addition on the grid for 3×10 seconds using a Sonopuls HD3200 sonicator with BB6 cup horn (Bandelin, Berlin) at 60% max output. Capsids were prepared for cryoET as described for neurons.

Data was collected on a Tecnai Polara (FEI, Eindhoven, The Netherlands) transmission electron microscope equipped with a GIF 2002 post-column energy filter (Gatan, Pleasanton, CA). Images were collected with a 2K×2K Multiscan CCD camera (Gatan). The microscope was operated at 300 kV and the pixel size was 0.81 nm at the specimen level. Tilt series were collected from −60° to 60°, with an angular increment of 2° or 3°. Defocus was measured along the tilt axis after each tilt and automatically maintained at −8 µm for isolated capsids and at −12 µm for neurons to gain phase contrast and to distinguish structures more accurately inside the cell. The total electron dose received at the specimen level was kept between 60 and 90 electrons/A^2^. The applied electron dose was kept proportional to 1/cosα of the tilt angle (α).

### Image processing

Tilted images were aligned using 10 nm gold beads as fiducial markers. Three-dimensional reconstructions were calculated with the software IMOD [85]. The volume of the reconstructions for visualization was typically 512×512×256 pixels, after the images obtained in the microscope (2048×2048 pixels) were down-sampled by a factor of four (IMOD). Subsequent processing steps were done using Bsoft [86]. Capsids were located in the original unbinned tomograms, and subvolumes with a size of 180×180×180 pixels were extracted. The orientation of all subvolumes was determined using a 22 Å resolution structure of the HSV1 capsid [87] as a template and the oriented subvolumes were averaged. Icosahedral symmetry was applied to the averages. Symmetrized averages were used as templates for the next iteration of orientation refinement. Three iterations were performed. The resolution of the averages was determined by Fourier shell correlation (FSC) using the 0.5 criterion, after splitting the data in two halves, calculating two separate averages and imposing icosahedral symmetry. To calculate the difference map of the two averages gray values were scaled to the same radial density maximum within the capsid and minimum just outside of the capsid. The difference of densities was then calculated by subtracting the capsids without tegument from the capsids with tegument. All capsid reconstructions were first scaled against the nuclear C-capsid reconstruction. Magnification differences up to 3.5% were detected and these were compensated for by creating up-scaled or down-scaled maps.

### Accession numbers

The following HSV-1 capsid maps have been deposited in the Electron Microscopy Data Bank (EMDB) at PDBe (http://www.ebi.ac.uk/pdbe/emdb/): EMD_1956, cytosolic C-capsids; EMD_1957, cytosolic A-/B-capsids; EMD_1958, nuclear A-capsids; EMD_1959, nuclear C-capsids.

The ID numbers for genes mentioned in the text (source: ncbi.nlm.nih.gov/gene) are: US9: Gene ID 2703452; UL17: Gene ID 2703388; UL25: Gene ID 2703377; UL35 (VP26): Gene ID 2703356; UL36: Gene ID 2703357; UL37: Gene ID 2703358.

## Supporting Information

Figure S1 **Types of intracellular progeny capsid found in axons.** (A) Cytosolic A-capsid. (B) Cytosolic B-capsid. (C) Cytosolic C-capsid. Bar: 100 nm.
(TIF)

Table S1 **Measured speeds and directionalities of capsids travelling inside axons at 16 h p.i..**
(DOC)
