# Supplementary figures and images for: Cryo Electron Tomography of Herpes Simplex Virus during Axonal Transport and Secondary Envelopment in Primary Neurons
Source: PLoS Pathog. 2011 Dec 15;7(12):e1002406. doi: 10.1371/journal.ppat.1002406 (PMC3240593; doi:10.1371/journal.ppat.1002406)

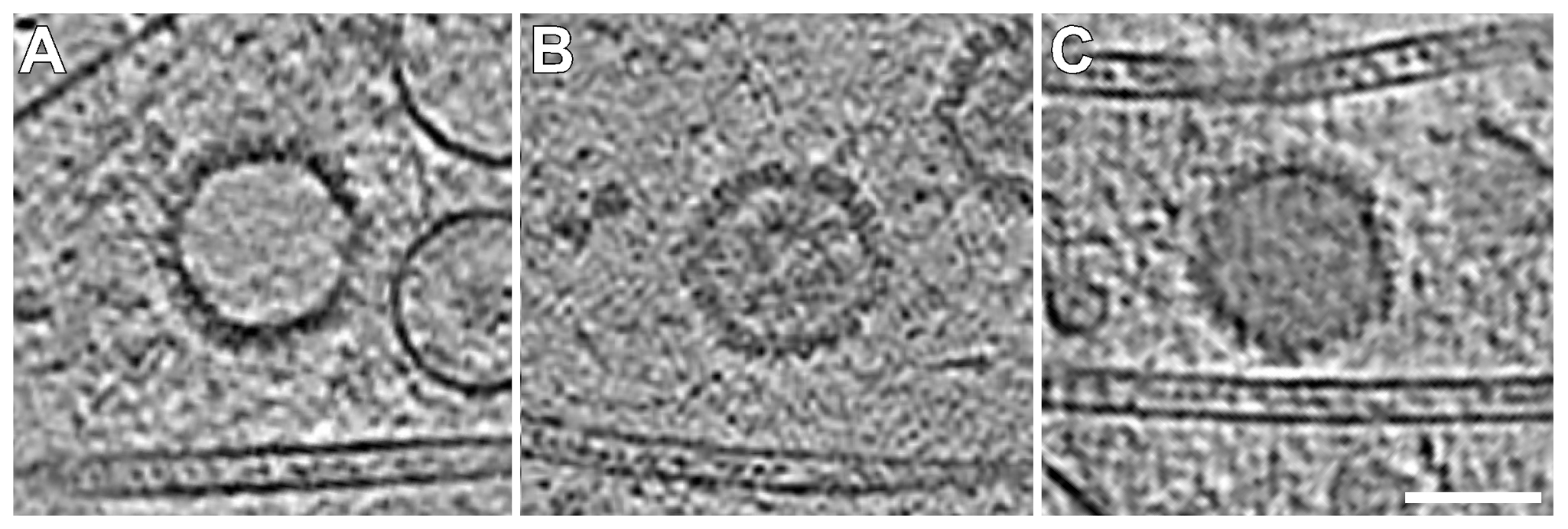

Supplement: Figure S1 — Types of intracellular progeny capsid found in axons. (A) Cytosolic A-capsid. (B) Cytosolic B-capsid. (C) Cytosolic C-capsid. Bar: 100 nm. (TIF) [file ppat.1002406.s001.tif]
