# Supplementary material for: Cryo Electron Tomography of Herpes Simplex Virus during Axonal Transport and Secondary Envelopment in Primary Neurons
Source: PLoS Pathog. 2011 Dec 15;7(12):e1002406. doi: 10.1371/journal.ppat.1002406 (PMC3240593; doi:10.1371/journal.ppat.1002406)
Supplement: Table S1 — Measured speeds and directionalities of capsids travelling inside axons at 16 h p.i.. (DOC) [file ppat.1002406.s002.doc]

**Table S1.** Measured speeds and directionalities of capsids travelling inside axons at 16 h p.i..

| **Capsids** | **p1** | **p2** | **p3** | **p4**^#^ | **p5**^#^ | **p6**^#^ | **p7**^#^ | **p8** | **p9** | **p10**^#^ | **p11** | **p12** | **p13**^#^ | **p14** | **p15** | **Total** |
| --- | --- | --- | --- | --- | --- | --- | --- | --- | --- | --- | --- | --- | --- | --- | --- | --- |
| Total run length (s) | 14 | 10 | 8 | 28 | 42 | 20 | 24 | 10 | 8 | 4 | 10 | 20 | 28 | 18 | 8 | **252** |
| Anterograde run (s) | 14 | 8 | 8 | 26 | 2 | 18 | 24 | 10 | 2 | 2 | 6 | 16 | 26 | 0 | 2 | **164** |
| Retrograde run (s) | 0 | 2 | 0 | 2 | 40 | 2 | 0 | 0 | 6 | 2 | 4 | 4 | 2 | 18 | 6 | **88** |
| % anterograde run | 100 | 80 | 100 | 93 | 5 | 90 | 100 | 100 | 25 | 50 | 60 | 80 | 93 | 0 | 25 | **65** |
| Total average speed (μm/s) | 0.9 | 2.1 | 3.4 | 3.5 | 3 | 1.7 | 1.5 | 2.2 | 1.5 | 1.3 | 0.9 | 1.3 | 2.2 | 1.2 | 3.4 | **2** |
| Average speed anterograde run (μm/s) | 0.9 | 2.3 | 3.4 | 3.7 | 6.1 | 1.8 | 1.5 | 2.2 | 1.8 | 1.1 | 1.1 | 1.7 | 2.3 | 0 | 3.7 | **2.4*** |
| Average speed retrograde run (μm/s) | 0 | 1.3 | 0 | 1.2 | 2.9 | 0.9 | 0 | 0 | 1.4 | 1.5 | 1.1 | 1.5 | 1.4 | 1.2 | 3.3 | **1.6*** |
| Maximum speed (μm/s) | 1.8 | 3.2 | 7.3 | 5.6 | 6.1 | 3 | 3.3 | 4.8 | 2.1 | 1.5 | 1.9 | 2.9 | 3.2 | 2.1 | 5.1 | **7.3** |

* Only moving particles considered.

^#^ Particle entered/left the field of view during the observation period.
